# Cancer survivorship at heart: a multidisciplinary cardio-oncology roadmap for healthcare professionals

**DOI:** 10.3389/fcvm.2023.1223660

**Published:** 2023-09-15

**Authors:** Irma Bisceglia, Maria Laura Canale, Nicola Silvestris, Giuseppina Gallucci, Andrea Camerini, Alessandro Inno, Massimiliano Camilli, Fabio Maria Turazza, Giulia Russo, Andrea Paccone, Raffaella Mistrulli, Leonardo De Luca, Stefania Angela Di Fusco, Luigi Tarantini, Fabiana Lucà, Stefano Oliva, Antonella Moreo, Nicola Maurea, Vincenzo Quagliariello, Giuseppina Rosaria Ricciardi, Chiara Lestuzzi, Damiana Fiscella, Iris Parrini, Vito Racanelli, Antonio Russo, Lorena Incorvaia, Fabio Calabrò, Giuseppe Curigliano, Saverio Cinieri, Michele Massimo Gulizia, Domenico Gabrielli, Fabrizio Oliva, Furio Colivicchi

**Affiliations:** ^1^ Integrated Cardiology Services, Cardio-Thoracic-Vascular Department, Azienda Ospedaliera San Camillo Forlanini, Rome, Italy; ^2^ Division of Cardiology, Ospedale Versilia, Azienda Usl Toscana Nord Ovest, Lido di Camaiore, Italy; ^3^ Unit of Medical Oncology, Department of Human Pathology in Adulthood and Childhood Gaetano Barresi, University of Messina, Messina, Italy; ^4^ Cardio-oncology Unit, Department of OncoHaematology, IRCCS Referral Cancer Center of Basilicata, Rionero in Vulture (PZ), Italy; ^5^ Department of Medical Oncology, Ospedale Versilia, Azienda Usl Toscana Nord Ovest, Lido di Camaiore, Italy; ^6^ Department of Oncology, Sacro Cuore Don Calabria Hospital (IRCCS), Negrar, Italy; ^7^ Department of Cardiovascular and Pulmonary Sciences, Catholic University of the Sacred Heart and Department of Cardiovascular Medicine, Fondazione Policlinico Universitario A. Gemelli IRCCS, Rome, Italy; ^8^ Cardiology Department, National Cancer Institute Foundation (IRCCS), Milan, Italy; ^9^ SC Patologie Cardiovascolari, Azienda Sanitaria Universitaria Giuliano Isontina (ASUGI), Trieste, Italy; ^10^ Department of Cardiology, G. Pascale National Cancer Institute Foundation (IRCCS), Naples, Italy; ^11^ Cardiology Unit, Department of Clinical and Molecular Medicine, Faculty of Medicine and Psychology, Sapienza University of Rome, Rome, Lazio, Italy; ^12^ Division of Cardiology, San Camillo-Forlanini Hospital, Rome, Italy; ^13^ Clinical and Rehabilitation Cardiology Unit, San Filippo Neri Hospital, ASL Roma 1, Rome, Italy; ^14^ Divisione di Cardiologia, Arcispedale S. Maria Nuova, Azienda Unità Sanitaria Locale-IRCCS di Reggio-Emilia, Reggio Emilia, Italy; ^15^ Cardiologia Interventistica, Utic, Grande Ospedale Metropolitano, Azienda Ospedaliera Bianchi Melacrino Morelli, Reggio Calabria, Italy; ^16^ UOSD Cardiologia di Interesse Oncologico, IRCCS Istituto Tumori "Giovanni Paolo II", Bari, Italy; ^17^ Cardio Center De Gasperis, Azienda Socio Sanitaria Territoriale Grande Ospedale Metropolitano Niguarda, Milan, Italy; ^18^ Unit of Medical Oncology, Papardo Hospital, Messina, Italy; ^19^ Centro Medico Esperia, Porcia (PN), Italy; ^20^ U.O.C. Cardiologia, Ospedale Garibaldi-Nesima, Azienda di Rilievo Nazionale e Alta Specializzazione “Garibaldi”, Catania, Italy; ^21^ Department of Cardiology, Hospital Mauritian Turin, Turin, Italy; ^22^ Department of Interdisciplinary Medicine, School of Medicine, University of Bari Aldo Moro, Bari, Italy; ^23^ Department of Surgical, Oncological and Oral Sciences, Section of Medical Oncology, Palermo University Hospital, Palermo, Italy; ^24^ Department of Oncology and Specialized Medicine, San Camillo-Forlanini Hospital, Rome, Italy; ^25^ Department of Oncology and Hemato-Oncology, University of Milan; Division of Early Drug Development, Istituto Europeo di Oncologia, IRCCS, Milan, Italy; ^26^ Medical Oncology Division and Breast Unit, Senatore Antonio Perrino Hospital, ASL Brindisi, Brindisi, Italy; ^27^ Fondazione per il Tuo cuore- Heart Care Foundation, Firenze, Italy; ^28^ Cardiologia 1- Emodinamica, Dipartimento Cardiotoracovascolare “A. De Gasperis”, Azienda Socio Sanitaria Territoriale Grande Ospedale Metropolitano Niguarda, Milan, Italy

**Keywords:** cancer survivors (CSs), cardiovascular disease (CVD), cancer therapy-related cardiovascular toxicities (CTR-CVT), cardiovascular risk factors (CVRF), reverse cardio-oncology, survivorship care

## Abstract

In cancer, a patient is considered a survivor from the time of initial diagnosis until the end of life. With improvements in early diagnosis and treatment, the number of cancer survivors (CS) has grown considerably and includes: (1) Patients cured and free from cancer who may be at risk of late-onset cancer therapy-related cardiovascular toxicity (CTR-CVT); (2) Patients with long-term control of not-curable cancers in whom CTR-CVT may need to be addressed. This paper highlights the importance of the cancer care continuum, of a patient-centered approach and of a prevention-oriented policy. The ultimate goal is a personalized care of CS, achievable only through a multidisciplinary-guided survivorship care plan, one that replaces the fragmented management of current healthcare systems. Collaboration between oncologists and cardiologists is the pillar of a framework in which primary care providers and other specialists must be engaged and in which familial, social and environmental factors are also taken into account.

## 1. Introduction

According to the National Cancer Institute Dictionary of cancer terms, “In cancer, a person is considered to be a survivor from the time of diagnosis until the end of life” (1). Due to screening programs, early detection and improved management of cancer, together with general population aging, cancer survivors (CSs) are a growing population that includes: 1) Patients cured and free from cancer (FFC) who are at risk of late-onset cancer therapy-related cardiovascular toxicity (CTR-CVT). 2) Patients with long-term control of not-curable cancers in whom CTR-CVT may need to be addressed: chronic cancer (CC) patients. In January 2022, more than 18 million Americans were considered to be CSs, ∼67% of whom were older than 65 years (2). This population also included ∼70,000 survivors of cancer during childhood and ∼49,000 survivors of cancer during adolescence. Europe has >12 million CSs, including ∼300,000 who survived a childhood cancer (3), many of whom are now at risk of developing cardiovascular toxicity (4). Emerging issues in cancer survivorship are also studied in the context of the “silver tsunami,” which refers to older CSs (5). In this group, the almost constant presence of co- morbidities may be complicated by late-onset CTR-CVT. An increased incidence of early-onset cancers, defined as cancers diagnosed in adults aged <50 years, has also been reported (6).

## 2. Cardiovascular risk in cancer survivors

Growing evidence of some common pathophysiologic mechanisms underlying cancer and cardiovascular disease (CVD) (7) (Table 1) has led to the development of “reverse cardio-oncology” (24). The premise is that by addressing modifiable common risk factors it is possible to reduce the risk of both CVD and cancer (25). A prevention-oriented model of cardio- oncology includes a pre-habilitation, habilitation and rehabilitation strategy to optimize physical fitness and cardiovascular health before, during and after cancer therapy, respectively (26–28). The newly published ESC Guidelines on Cardio-Oncology (29) emphasize the role of the “baseline CV risk stratification proformas” (30), provided by the Cardio-Oncology Study Group of the ESC and the International Cardio-Oncology Society, and the dynamic variability of the risk. An assessment of the baseline CV risk should be part of a personalized approach in CSs and the first step of every clinical risk predictive model for these patients. Precision cardio-oncology, providing individualized algorithms based on CV risk, cancer type and cancer treatments is expected to be a valuable tool in survivorship care. Furthermore genetically-defined risk factors, such as polymorphisms in drug-related damage genes, could add value to risk scores in the next future. The use of more sophisticated models relies upon dedicated datasets obtained by the collection of systematic and, if possible, prospective data optimized using artificial intelligence tools (31).

**Table 1:** Shared risk factors between cardiovascular disease and cancer: pathogenesis, underlying biological mechanisms, tumours related to risk factors.

Factors	Biological pathways/mediators	Pathophysiologic mechanisms underlying cancer and CVD risk	Related tumours	References
Epigenetic instability	Somatic JAK2 mutations	Uncontrolled cell proliferation		Wang W, et al. Circ Res 2018 (8)
	Imbalance in reactive oxygen species	Lipid peroxidation and DNA damage		Balzan S, et al. Life Sci 2018 (9)
Diabetes mellitus	Insulin/IGF activates many pathways: Ras/MEK/ERK, MAPK, Pl3K/AKT/mTOR	Neo-angiogenesis cancer cell proliferation, metastasis	Colorectal, hepatocellular, gallbladder, breast, endometrial, and pancreatic cancers	Di Fusco SA, et al. Int J Cardiol 2022 (7)
				Pearson-Stuttard J Cancer Epdemiol
				Biomarkers Prev 2021 (10)
Hypertension	Renin-angiotensin system	Angiotensin II stimulates the release of VEGF, a mediator of tumor angiogenesis whose levels are increased in hypertension.	Renal cell carcinoma, esophageal squamous cell carcinoma	Felmeden DC et al. Am J Hypertens 2003 (11)
				Christakoudi S, et al. Int J Cancer. 2020 (12)
	Hyperactivation of the sympathetic nervous system	DNA damage and p53 suppression through β- adrenoceptors xpressed on cancer cells		Hara MR, et al. Nature 2011 (13)
	GRK4	Enhanced GRK4 activity reduces renal sodium excretion, may lead to hypertension, and promote BC cell proliferation		Yue W, et al. 2021 (14)
Dyslipidemia	Hypercholesterolemia	Impaired immunosurveillance mediated by natural killer and dendritic cells has been documented	Prostate cancer	Herber DL, et al. Nat Med 2010 (15)
				Giunchi F et al. Eur. Urol. Oncol. 2019 (16)
				Sousa AP et al. Front Cell Dev Biol.2022 (17)
		Modulation of multipotent hematopoietic stem and progenitor cell functions		Li C, et al. Nutr Res 2016 (18)
Obesity	Adipokines, including adiponectin, leptin, and resistin, that are up-regulated in obesity	Adipokines induce miR- 21, a tumorigenesis regulator, and may impact on hypertension	Oesophageal, stomach, liver, pancreas, gallbladder, ovary, thyroid, kidney cancer, postmenopausal breast cancer, endometrium cancer, advanced prostate cancer and multiple myeloma	Jasinski-Bergner S, et al. Obes Facts 2019 (19)
				World cancer research fund international.
				Continuous update project expert report 2018 (20)
Smoking Factors	Pro-inflammatory pathways elicited by cigarette smoke and secondhand smoke through irritants, carcinogens, and oxidative stress.	Endothelial dysfunction, increased release of inflammatory cytokines, activation of NF-kB, increased expression of adhesion molecules, MMP activation, and reduced MMP inhibitors.	Lung cancer, bladder cancer Related tumours	Morris PB et al. J Am Coll Cardiol. 2015 (21)
				Koene RJ et al. circulation 2016 (22)
	Nicotine. Biological pathways/mediators	Pathophysiologic mechanisms underlying cancer and CVD risk		U.S. department of health and human services PHS: CDC publication; 2004 (23)

JAK2, Janus kinase 2; VEGF, vascular endothelial growth factor; GRK4, G-protein-coupled receptor kinase 4; CVD, cardiovascular disease; NF-kB, nuclear factor kappa B; MMP, matrix metalloproteinases.

### 2.1. FFC-Childhood, adolescent and young adult survivors

Thirty years after treatment, as many as one in eight survivors of childhood cancer treated with anthracyclines and chest radiotherapy (RT) will develop a life-threatening CVD, and the likelihood increases as survival progresses. Exposure to doxorubicin at doses ≥250 mg/m^2^ and to ≥15 Gy of chest RT is considered as a high-risk condition, as it increases the relative risk of heart failure as well as pericardial and valvular disease by 2- to 5-fold compared to non-exposure (32). The recent reduction in the incidence of coronary artery disease (CAD) in children with cancer is due to the greater attention paid to limiting cardiac exposure to RT (33). Cancer treatment is also associated with the risk of developing comorbidities, and 40% of childhood, adolescent and young adult CSs have multiple comorbidities 10 years after the index date. Childhood, adolescent and young adult CSs also have an increased risk of cardiomyopathy, stroke, premature ovarian failure, chronic liver disease, thyroid disorders, diabetes, hearing loss and renal failure (34).

### 2.2. FFC-adult survivors

Older women with breast cancer have a higher risk of dying from CVD than controls. Thus, according to the “multiple-hit” hypothesis (35) the main effort should be directed at reducing a high CV risk profile worsened by cancer treatment. An assessment of the CVD risk within the first year after completion of cardiotoxic cancer therapy can identify CSs who require long-term surveillance. Patients with high/very high baseline CVD risk factors and those who develop ventricular dysfunction at the end of treatment are at high risk of CVD during the first 2 post-treatment years (“early” high risk), while patients treated with high doses of anthracyclines plus RT or with poor control of cardiovascular risk factors (CVRFs) are at high risk of late CVD (“late” high risk). The ESC guidelines on CO provide recommendations for survivors of childhood and adolescent cancer and for adult CSs (29) (Table 2).

**Table 2:** Recommendations for asymptomatic CSs (Ref. 29).

Asymptomatic childhood/ adolescent CSs	Asymptomatic adult CSs
Educate adult survivors of childhood or adolescent cancer treated with anthracyclines, mitoxantrone and/or thoracic RT that includes the heart on their increased CV risk	Perform annual CV risk assessment that takes into account ECG, NP, and CVRF correction in CSs who have received RT or cardiotoxic therapies
Perform annual screening for modifiable CVRFs in adult survivors of childhood or adolescent cancer treated with anthracyclines, mitoxantrone and/or RT that includes the heart	Plan to re-evaluate the risk of CV toxicity 5 years after treatment
Communicate the increased risk status of patients to all healthcare providers	Perform echocardiographic follow-up at years 1, 3 and 5 after the end of cardiotoxic treatment and every 5 years thereafter in very high** and early high-risk** patients; follow-up at 5 years and every 5 years thereafter in late high-risk** patients, follow up every 5 years in moderate risk** patients
Assess risk in female survivors of childhood or adolescent cancer before pregnancy or during the first trimester	Perform non-invasive testing for CAD, every 5 to 10 years, in asymptomatic patients who have received >15 Gy MHD, starting 5 years after RT
Perform echocardiographic monitoring every 2 years in adults at high risk* and every 5 years in adults at moderate risk*	Perform a carotid doppler echo five years after the end of RT head/neck treatment, and then every 5–10 years thereafter

CAD, coronary artery disease; CSs, cancer survivors; CV, cardiovascular; CVRFs, cardiovascular risk factors; ECG, electrocardiogram; MHD, mean heart dose; NP, natriuretic peptides; RT, radiotherapy.

* **Very high risk**: RT dose (Gy MHD) > 25, total cumulative doxorubicin dose (mg/m^2^) ≥ 400; combination therapy: RT dose >15 + total.

cumulative doxorubicin ≥100.

* **High risk**: RT dose (Gy MHD) > 15 to 25, total cumulative doxorubicin dose (mg/m^2^) 250 −399; combination therapy: RT dose (Gy MHD).

5-15 + total cumulative doxorubicin ≥100.

* **Moderate risk**: RT dose (Gy MHD) 5–15, total cumulative doxorubicin dose (mg/m^2^)100–249; combination therapy: RT dose <5.

+ total cumulative doxorubicin ≥100.

** **Very high risk**: very high baseline CV toxicity risk pre-treatment, doxorubicin ≥400 mg/m^2^, RT > 25 Gy (MHD), combination therapy RT > 15–25 Gy MHD.

+ doxorubicin ≥100 mg/m^2^.

** **Early high risk** (<5 years after therapy): High baseline CV toxicity risk, symptomatic or asymptomatic moderate-to-severe cancer therapy-related cardiac dysfunction during treatment, doxorubicin 250–399 mg/m^2^, high risk haematopoietic stem cell transplantation.

** **Late high risk**: RT > 15–25, combination therapy: RT 5–15 Gy MHD + doxorubicin ≥100 mg/m^2^; poor control of CVRFs.

** **Moderate risk**: Moderate baseline CV toxicity risk, doxorubicin 100–249 mg/m^2^, RT 5–15 Gy MHD, combination therapy: RT < 5 Gy MHD.

+ doxorubicin ≥100.

### 2.3. Chronic cancer patients

New treatment approaches are changing the natural history of many cancers, including metastatic breast (36), prostate (37), lung cancer (38), lymphomas (39) and other common cancers, while also increasing the risk of CVD-related death or disabilities (40, 41). A decline in adherence to prescribed cardiovascular drugs has also been reported (42).

## 3. Cardiovascular monitoring of long-term survivors

### 3.1. Biomarkers

Cardiac troponin and natriuretic peptide are the most extensively evaluated cardiac biomarkers. Both have been used to investigate the cardiac toxicity of anthracyclines and HER2-inhibitors, whereas their utility in patients treated with other anticancer drugs (43, 44) and the value of their routine use in patients undergoing potentially cardiotoxic treatment are thus far unclear. According to the European Society for Medical Oncology guidelines, baseline measurements of cardiac biomarkers should be considered for high-risk patients (i.e., those with preexisting significant CVD) and those receiving high doses of cardiotoxic chemotherapy such as anthracyclines (45). The recently published ESC guidelines on cardio-oncology recommend the use of natriuretic peptide and/or troponin before anticancer therapy in all cancer patients at risk of CTR- CVT if these biomarkers are going to be measured during treatment to detect CV toxicity (class I, level C recommendation) (29). Baseline biomarkers may indeed allow the identification of those patients most likely to benefit from cardio-protective therapy. A potential prognostic role of troponins and natriuretic peptides has also been investigated (46, 47). More recently, exercise capacity and maximal oxygen consumption have emerged as potential biomarkers in cardio-oncology (48, 49).

#### 3.1.1. The controversial role of cardiac biomarkers in the long-term follow-up of asymptomatic cancer survivors

The significantly higher odds of an elevated high- sensitivity cardiac troponin level in CSs reflects the high risk of subclinical myocardial damage. Accordingly, its elevated levels could identify patients who need personalized monitoring and tailored strategies to mitigate the CV risk. Abnormal natriuretic peptide values identify CSs exposed to cardiotoxic therapy and at increased risk of future cardiomyopathy despite a preserved left ventricular ejection fraction. The ESC guidelines recommend an annual risk assessment, including an electrocardiogram and the measurement of natriuretic peptide levels, in adult CSs whose treatment consisted of a cardiotoxic cancer drug or chest RT (29) (Table 3).

**Table 3:** Summary of studies on the role of biomarkers in cancer survivors

Study	Population	Biomarker	Results
Florido et al. 2019 ARIC study (50) (prospective community-based cohort study)	12,414 participants 25% had incident cancer over a median 13.6 years of follow-up.	cTn and NT-proBNP	Participants with a high hs-cTnT had an increased risk of CAD, fatal CAD, total mortality, and HF. Participants with a detectable hs-TnT level 6 years apart had an increased risk of subsequent CAD, HF, and death; CS had a significantly higher odds of an elevated hs-cTnT
Michel et al. 2020 (51) (meta-analysis)	5,691 cancer patients receiving potentially cardiotoxic therapies (61 studies)	cTn and NT-proBNP	Significant association between Tn elevation and LV systolic dysfunction, with a positive predictive value of 52% and a negative predictive value of 93%.
Michel et al. 2020 (52) (meta-analysis)	An update that includes an additional study	cTn and NT-proBNP	Two-fold increase in LV systolic dysfunction with elevated BNP/NT- pro BNP
Dixon et al. 2021 (53) (St. Jude Lifetime Cohort study)	1213 adults ≥10 years after a childhood cancer diagnosis; 786 were treated with anthracycline chemotherapy	cTn and NT-proBNP	Abnormal NT- proBNP levels were determined in 25% of cases but only 0.4% had abnormal Tn values. Survivors who had received higher doses of anthracyclines or chest RT had at least a 3-fold higher risk of abnormal NT- proBNP
Peel et al. 2014 (48) (retrospective)	27 studies including breast cancer patients after adjuvant therapy	VO2 max	Mean VO2max was 25% lower in breast cancer patients than in healthy, sedentary women
Ness et al. 2020 (49) (cross-sectional)	1,041 CSs and a control group of 285 individuals	VO2 max	Survivors had a lower mean peak oxygen uptake than controls

cTn, cardiac troponins; NT-proBNP, N-terminal pro BNP; LV, left ventricle; BNP, brain natriuretic peptide; VO2max, maximal oxygen uptake; CAD, coronary artery disease; HF, heart failure; CS, cancer survivors; RT, radiotherapy.

### 3.2. Imaging

Imaging surveillance in CSs allows the early identification of CTR-CVT, as also reported in the recent ESC guidelines (29). Among multiple cardiac imaging techniques used in patient surveillance, the most accessible is echocardiography, although cardiac magnetic resonance and coronary computed tomography may be as valuable as echocardiography in selected cases. The monitoring of myocardial function during the post-treatment phase relies on left ventricular ejection fraction evaluation whereas during oncologic treatment its evaluation should be coupled with assessments of more sensitive functional parameters (e.g., global longitudinal strain) (54). Besides an annual clinical evaluation, moderate risk patients should have an echocardiogram every 5 years and high/ very-high risk patients at 1, 3 and 5 years and then every 5 years thereafter except in patients with overt symptomatology (29). With its ability to assess cardiac structure and function and to provide myocardial tissue characterization, multi-parametric cardiac magnetic resonance has a preeminent role in the assessment of CVD in cardio-oncology (55). However, its role in CSs is limited although the use of non-contrast cardiac magnetic resonance protocols was shown to be of value in particular settings (55). Moreover, coronary computed tomography may be useful in CSs to evaluate ischemia or myocardial dysfunction (56) and to detect subclinical atherosclerosis, quantified using the coronary artery calcium score. This well-known imaging biomarker is an indicator of the extent of coronary calcification and has independent prognostic value. An advantage of coronary computed tomography is that it may allow adequate planning of structural interventions in patients with complex valve diseases, which are frequently responsible for heart failure in this population (56).

## 4. Lowering the cardiovascular risk in cancer survivors

### 4.1. Non-pharmacological strategies

**Figure 1:**
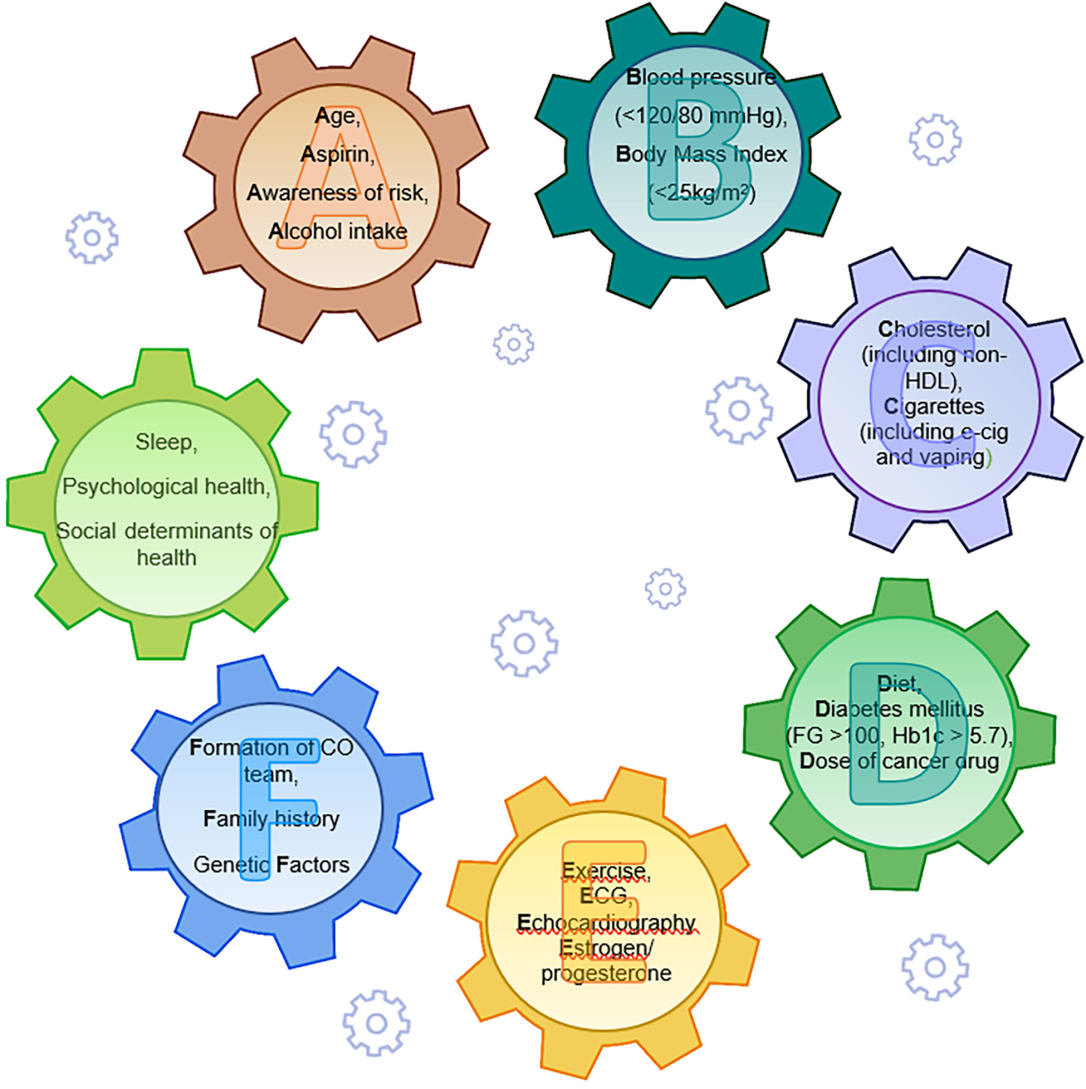
Life's essentials 8 in preventive cardio-oncology: the ABCDEF approach with behavioral components, ideal parameters and psychological/sociological issues (in green) working as interactive gears [modified from brown SA (57) and Lloyd-Jones DM et al. (58, 59)]. Non-HDL, non high density lipoprotein cholesterol; FBG, fasting blood glucose; HbA1c, hemoglobin A1c; CO, cardio-oncology.

Prevention in cardio-oncology is based on the ABCDEF approach (57) addressing the seven factors of AHA Life's Simple 7 (58). In the cardio-oncology perspective, “A” stands for age (a known enhancer of CV risk), aspirin (a widely used drug in CVD), “awareness of risk” (a pivotal point in prevention) and alcohol intake, “B” stands for Blood Pressure and Body Mass index, “C” stands for cigarette use and cholesterol, “D” for diet, diabetes and dose of cancer therapy, “E” for exercise, estrogen/progesterone, echocardiography and other diagnostic tools, F for “formation of cardio-oncologic team”, family history and genetic factors. Psychological and sociological issues have been recently added as components of a healthy life (59) (Figure 1). “Awareness of risk” highlights the importance of an increased awareness of CTR-CVT, and “Formation of a cardio-oncology team” the need for a truly multidisciplinary approach to cancer patients. The same parameters for cardiovascular health applied to the general population serve as references for preventative, non-pharmacological interventions to reduce the CV risk in CSs. The 2022 ESC guidelines state that “dietary patterns with a high intake of vegetables/fruits and whole grains are associated with lower rates of mortality and cancer recurrence than is the case for diets with a high content of refined grains, processed and red meats, and high-fat dairy products” (29). Regular physical activity and exercise have a documented favorable impact on CVD (60) and on NP levels, including an “exercise-induced sacubitril-like effect” (61), whereas unhealthy diets, inactivity and obesity are associated with cancer recurrence risk and a shortened survival, as shown in breast cancer survivors (62, 63). Physical activity pre-diagnosis is important in reducing cancer-specific and all-cause mortality, even more so is physical activity post diagnosis (64, 65).

### 4.2. Pharmacological strategies

In the primary prevention of CVR-CVT, randomized clinical trials have shown a beneficial effect of beta-blockers/angiotensin-converting enzyme inhibitors/angiotensin-receptor blockers, administered during anthracycline treatment with or without trastuzumab, on left ventricular function decline, but in all trials the patient population was small and heterogeneous so a general recommendation cannot be made (66–68). As a “universal” strategy of cardioprotection for all patients is so unfeasible, a careful triage is needed to deliver cardioprotective treatment only to high-risk patients (69) as indicated also by guidelines (29). Cardioprotective pharmacological treatment of CSs must aggressively address pre-existing CVRFs and cancer-therapy-induced derangements in cardiometabolic pathways and should be combined with non-pharmacological interventions (70). The intersection of poor adherence to the cardiac medications necessitated by cancer treatment, chemotherapy-induced metabolic derangements (e.g., weight gain) (71) and cancer-treatment-induced reductions in cardiorespiratory reserve exemplify the dangerous combination of “hits” that may translate into a clinical cardiac event (35).

A more specific strategy would imply a customized treatment targeted to the side effect of cancer therapy, e.g. a rigid control of hypertension in patients treated with drugs affecting blood pressure such as tyrosine kinase inhibitors and VEGF inhibitors; a lipid-lowering therapy in patients treated with hormone therapy for prostate and breast cancer that often induce dyslipidemia (72); optimal treatment of diabetes in patients treated with PEG-L-asparaginase, corticosteroid-containing regimens, targeted therapies causing derangement in molecular pathways involved in glucose homeostasis, immune checkpoint inhibitors inducing immune-related adverse events such as type 1 diabetes. On the other hand, a tumour-oriented strategy would imply an aggressive treatment of the shared risk factors between cancer and CVD (e.g. obesity in breast cancer**).**

## 5. Management of *older* cancer survivors

The majority of CSs are ≥65 years of age (2) and by 2040 the proportion of patients aged 75 years or older is expected to reach 50% (73). Cardiovascular vulnerability in older CSs involves the entire CV system, as the frailties and multi- morbidities that often accompany aging interact with the consequences of cancer treatment (74). In a study of 31 million Medicare beneficiary patients, 67% had multi-morbidities, and the rate increased significantly with age (75). The individuals at highest risk for CV complications are elderly CSs previously treated with anthracyclines and/or chest RT. Most studies thus far have focused on lymphoma and breast cancer survivors. In a study based on data from the SEER program, which included 3,910 patients >65 years of age who had been diagnosed with diffuse large B-cell lymphoma and treated with doxorubicin-based chemotherapy, the risk of congestive heart failure/myocardiopathy within 6 months and 3 years after diagnosis was higher than in controls. The risk of acute myocardial infarction at both time points was also increased (76). However, studies in breast cancer survivors have produced inconsistent results (77, 78). In the elderly, both oncologic treatment and long-term surveillance of CTR-CVT may be complicated by poor adherence to treatment and follow-up programs. Higher rates of disability, geriatric syndromes, vulnerability and frailty are observed in elderly CSs after chemotherapy with curative intent (79). Cancer-related cognitive impairment has been determined in 40% of older survivors in the 18 months after diagnosis (80). An emerging area of interest is the impact of cancer on “resilience,” defined in this context as an ability to respond to stress that could prevent or delay the progression of multi-morbidity, disability and cognitive impairment in elderly CSs (81). Nonetheless, the cardio-oncologist should monitor all stressors in elderly CSs to prevent or at least reduce their impact on a vulnerable CV system. Four key points emerged at the U13 conference of the Cancer and Aging Research Group: 1) survivorship care is a process that continually evolves to meet the needs of older adults; 2) older adult CSs have unique needs, and care plans should be tailored to meet those needs; 3) a multidisciplinary team is essential for structuring survivorship care for older adults and 4) patient advocacy must be encouraged throughout the cancer care pathway (82).

## 6. Management of cancer patients treated with chest radiotherapy

### 6.1. Cardiovascular risks associated with radiation therapy

Among the late effects of RT, fibrosis involving all cardiac structures is of special importance (83). Furthermore, many chemotherapeutic agents cause oxidative stress, increasing the risk of RT-induced heart disease (RIHD) (84). The damage is chronic and usually becomes clinically evident after several years, increasing steadily over time (85, 86). However, the incidence of clinically relevant RIHD does not increase linearly and does not reach a plateau (87). The risk of RIHD is proportional to the radiation burden to the whole heart and to individual structures and differs depending on the setting. The RT fields used in breast cancer involve mostly the left anterior descending coronary artery and the left or right ventricle (depending on the irradiated breast) (88, 89). Not surprisingly, the risk of clinically relevant heart disease is higher for patients with left breast RT and is represented by CAD (90, 91). As the irradiated heart often behaves like a denervated heart, CAD may be under-diagnosed because silent ischemia is more frequent than in the general population (92). In RT for lung cancer, the risk of cardiac damage is primarily influenced by the site of the tumor (upper vs. lower lobes) and the type of RT: the risk associated with left-sided tumors is higher with dose-intensified three-dimensional conformal RT + intensity-modulated RT but not with stereotactic body RT (93). For several reasons, the most critical population at risk of RT-induced heart disease consists of patients cured of mediastinal lymphoma. The young age at the time of treatment and the high rate of complete remission are associated with an extended survival of at least 40 years that increases the probability of late CTR-CVT. Moreover, up to the 1990s, RT was delivered with extended and mantle fields and at high doses. While new modes of RT delivery have since been developed, patients successfully treated before their implementation are now in their 50s and 60s and are at very high risk of cardiac damage (94, 95). The most common phenotype of RIHD is CAD involving the left coronary artery with ostial, main trunk or LAD lesions, or the right coronary artery at the proximal level (96–98). RT-induced valvular heart disease has a high fibrotic and calcific component, rapidly progresses to severe forms and mostly involves the aortic valve (99, 100). Acute pericarditis is relatively common during or shortly after chest RT. However, it may evolve toward a chronic constrictive or effusive-constrictive pericarditis. These patients have a worse outcome than those with other types of constrictive pericarditis (101, 102). Autonomic dysfunction leading to an increased resting heart rate, impaired heart rate variability, atrial fibrillation and atrio-ventricular block (103) are other phenotypes of RT-induced heart disease.

### 6.2. Follow-up planning

The need for a lifelong surveillance in CSs treated by chest RT is well recognized, including in the recently released ESC guidelines, which recommend an annual CV risk assessment that includes ECG, measurement of NP levels and an assessment of CVRFs, echocardiography in asymptomatic high-risk adult CSs starting 5 years after RT (delivered to a volume that included the heart) and every 5 years thereafter, and non-invasive screening for CAD every 5–10 years in asymptomatic patients who received 15 Gy of median heart dose, starting 5 years after radiation (29). Screening for CAD may consist of physical or pharmacological stress tests, CT scan with assessment of the calcium score, coronary CT, or even coronary angiography (104–106). Of these, the calcium score and coronary CT have been used in many studies (105, 106). The choice should be made according to the availability of each test and according to the needs of the patient.

### 6.3. The management of FFC survivors

The evolution of RIHD can be accelerated by the presence of CVRFs. Chest RT is an independent risk factor for CAD (107–109). To prevent the progression of cardiac damage, patients should be encouraged to maintain a healthy lifestyle (110–112). Statins should be prescribed to patients with even mild dyslipidemia (113) and ACEI may have favorable effects on RT-induced fibrosis, both in the heart and in the lungs (114). Inappropriate sinus tachycardia secondary to autonomic dysfunction can be treated with BB, non-dihydropyridine calcium channel blockers and, in the rather frequent case of a hypotensive patient, ivabradine (115). Surgical treatment of RIHD is often challenging. Because many patients with valvular heart disease have CAD and/or pericardial disease, any intervention in those with extensive calcifications, mediastinal fibrosis or pericardial adhesion (frequently observed after mediastinal RT) is more difficult, and the short- and long- term outcomes accordingly worse (116–118). A multidisciplinary approach is recommended to assess and define the surgical risk in CSs with severe valvular heart disease (29). Percutaneous interventions are preferred over surgery whenever possible, even if the clinical outcome is still worse in patients with RIHD than in those who did not receive RT (119–121).

## 7. Creating a roadmap to improve monitoring strategies

**Figure 2:**
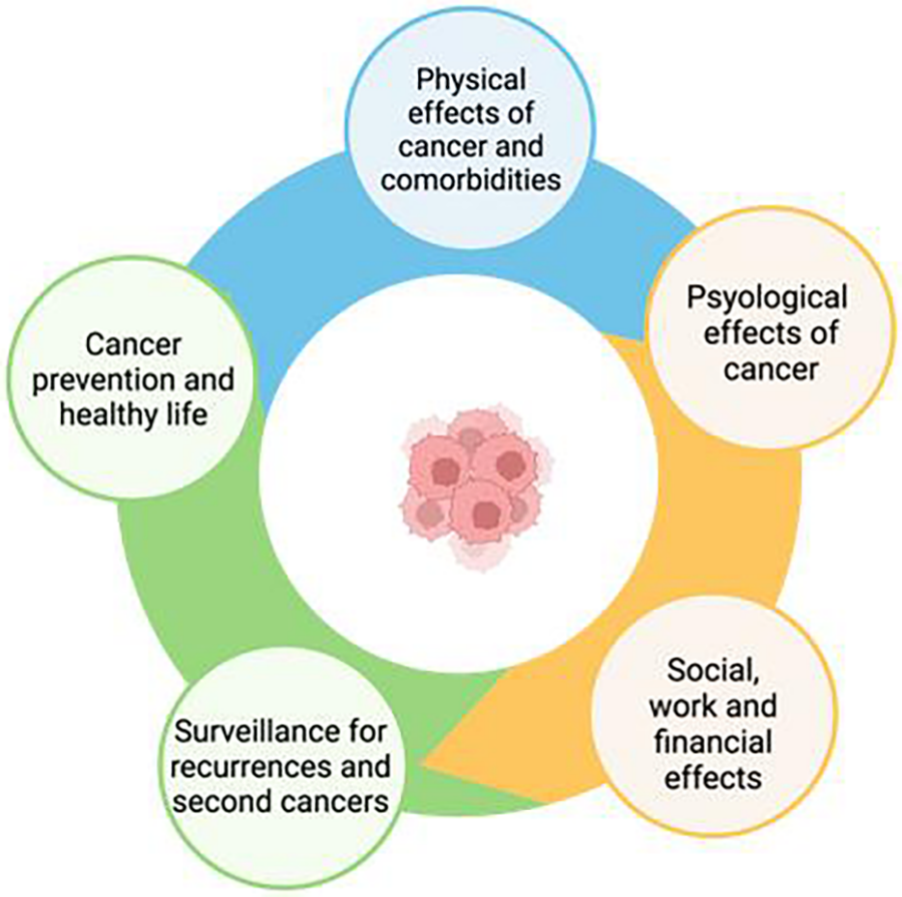
Areas to be addressed in survivorship care plans (modified from Vaz-luis et al. 125).

**Figure 3:**
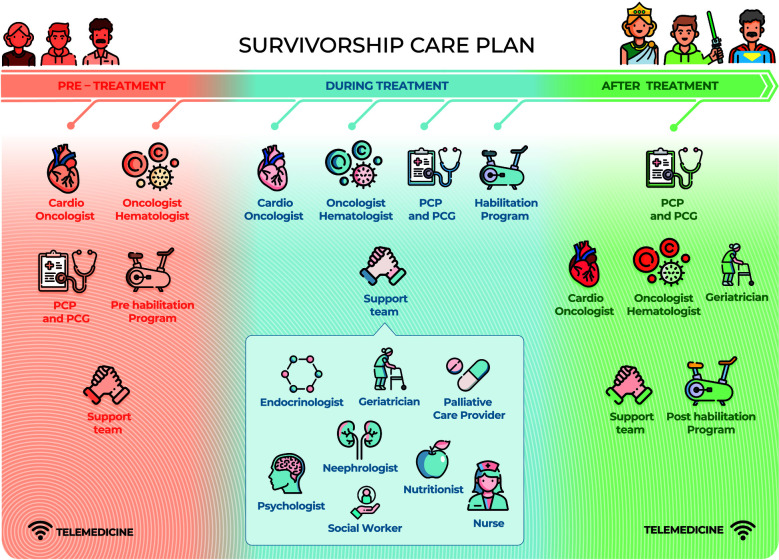
A comprehensive multidisciplinary care plan supporting every cancer survivor. Cardio-Oncologists and Onco-Hematologists guide the pre-treatment *red* phase while PCP, PCG and Pre-hab have a helping role. Treatment *blue* phase is more challenging, therefore Cardio- Oncologists, Onco-Hematologists, PCP, PCG, and Rehabilitation team all together lead the way in a close collaboration. PCP, PCG and geriatricians are in charge in the long post-treatment *green* phase getting advices from Cardio-Oncologists, Onco-Hematologists, and Post-hab team. A support team is always backing-up. Patients are the core of the survivorship plan and they become super-powered during the journey (Patient Empowerment). Telemedicine will facilitate patient-healthcare providers' interactions in each phase. PCP, Primary Care Provider; PCG, primary CareGivers.

Survivorship care plans are essential to ensure the quality of care for CSs. As has often been stated, “Failing to plan is planning to fail” (122). The psychological symptoms and physical disorders reported by CSs, such as fatigue, pain, anxiety, insomnia, depression, fear of recurrence, impaired cognitive and sexual function, difficulty in returning to work and a decreased quality of life, can persist for years after the end of treatment (123). Many CSs experience the transition phase from the end of treatment to follow-up as a time of loneliness and lack of support, with >60% of patients complaining of an average of five unmet needs in the first year (124) and continuing to various degrees thereafter. Optimization of the care of CSs was considered in the recent expert consensus of the European Society for Medical Oncology, in which five areas that must be systematically addressed in care plans from the beginning were identified (125) (Figure 2). An ideal survivorship care model requires collaboration between cardiologists and oncologists. Both specialists are the pillars of the framework and must engage a multidisciplinary team who must be fully aware of the needs and vulnerabilities of CSs. In the difficult task of survivorship care, digital care models have attracted interest because of their ease of access in various settings, but barriers that limit their implementation still remain. A recent review proposed several key points required for achieving integrated, individualized care, beginning at cancer diagnosis and continuing during treatment and follow-up phases (126) (Table 4). A prerequisite is the identification of clinical resources and their potential for deployment over time. Telemedicine may be of particular value in this context. The advantages of telemedicine were well demonstrated during the COVID-19 pandemic, as virtual platforms proved highly useful for multidisciplinary specialist meetings and video consultations with patients (127, 128). A recent literature review summarized the evidence on the effectiveness and implementation of telemedicine in the post treatment phase of cancer (129). However, the absence of supporting guidelines, inadequate reimbursement methods as well as a lack of training and education have hindered the full use of telemedicine in CSs and in other settings. We propose a comprehensive multidisciplinary approach along the whole CSs' pathway (Figure 3).

**Table 4:** A roadmap for the care of cancer survivors: who, when and how.

	Before treatment	During treatment	After treatment
Oncologist hematologist	Risk stratification for CTR-CVT.	Checking for early signs/symptoms of CTR-CVT	Specific search for CTR-CVT during follow-up.
Cardio-oncologist	Creating a customized proactive CO plan.	Planned imaging and visits	CO rehabilitation programs
	Actions to reduce CTR-CVT and general CV risk factors	CO habilitation during cancer treatment	Long-term CO follow- up according to the baseline risk for CTR- CVT and the experienced toxicity
	Pre-habilitation		
Patient caregivers (PCG)	CTR-CVT awareness lifestyle modifications	Treatment compliance. Early report of sign and symptoms.	Maintenance of lifestyle improvements social engagement long-term compliance with CO follow-up
		Daily support.	
Primary care provider (PCP)*	Shared action to reduce CTR-CVT and general CV risk factors.	Counseling and daily support	Long-term global health management focus on CVRF
Support team (ST)*: psychologist, geriatrician, nutritionist, palliative care provider, nurse, subspecialists, social workers	Management of anxiety Specific diet	Nutritional support management of anticancer drugs’ side effects and of pain	Psychology issues (school, work, social) long-term toxicity
	Reduce medications, management of comorbidities		

## 8. Cardio-oncologic rehabilitation

A supervised exercise program, including high-intensity interval training, is safe and well-tolerated in cancer patients, mitigates their risk of CTR- CVT and improves their cardiorespiratory fitness, as also stated in the 2022 ESC guidelines (29). In addition to improving the quality of life and cardiorespiratory fitness (CRF), cardiac rehabilitation reduces and prevents mortality and morbidity in patients with CVD. CRF is an independent predictor of all-cause mortality after adjusting for traditional risk factors (130). In 2019, the American Heart Association proposed the Cardio-Oncology rehabilitation program as a multidisciplinary approach to CV rehabilitation in CSs (131). Cardio-Oncology rehabilitation is based on the well-established cardiac rehabilitation programs for non-cancer cardiology patients and employs the multi-modality approaches used in those programs, including a complete structured exercise regimen together with nutritional counseling, psychological support and CV risk assessment. The aim of rehabilitation is to support cancer patients in their efforts to maintain a healthy and active life before, during and after treatment. Jones et al. found that breast cancer patients have markedly impaired cardiorespiratory fitness across the entire survivorship continuum and that the VO2 peak may be an independent predictor of survival in metastatic disease (132). CRF determination is a key aspect of the Cardio-Oncology rehabilitation program: maximal cardiopulmonary exercise testing serves as the gold standard and the 6-minute walking test as an alternative (133). The cancer patients that benefit the most from Cardio-Oncology rehabilitation are high-risk patients with CVRFs and patients who received high-risk treatment, such as high-dose cardiotoxic chemotherapy or thoracic RT. Survivors of childhood cancers may likewise benefit from rehabilitation. Some observational studies and meta-analyses of the effects of exercise in cancer patients are summarized in Table 5.

**Table 5:** Observational studies and meta-analyses of the effects of exercise in cancer survivors.

References	Population	Methods/Type of exercise	Results
Palomo A. et al.	4,015 patients with early BC	Patients’ activities were divided into quartiles according to Met-h per week (<2.5 Met-h/w, 2.5–8.625 Met-h/w, 8.625–18 Mets, >18 Met-h/w)	Exercise training of about 18 Met-h/w lowered the risk of CV events and coronary death during a median follow-up of 12.7 years
Okwuosa			
OkwuosaTM et al. (observational study) (64, 134)			
Williamson T. et al. (observational cohort study) (135)	442 patients with CVD and preexisting cancer of any type	12-week exercise- based CORE program	Patients who were able to complete the CORE program and achieved at least a 1.5-Met improvement in CRF had a significantly better overall survival
Jones LW et al. (prospective study) (136)	2,974 patients with early BC	Patient's recreational physical activities	Adherence to exercise guidelines (>9 Met-h/w) was associated with a 23% reduction in cardiovascular risk
Lahart IM. et al. (meta-analysis) (137)	22 prospective cohort studies (123, 574 patients)	Patient's recreational physical activities	Patients who reported high lifetime recreational pre-diagnosis physical activity levels (>8 Met-h/w) had a significantly lower risk of all- cause and BC-related death
Foulkes JS. Et al. (randomized study) (138)	104 women with early BC	Randomized to 3–4 days/week of aerobic and resistance exercise training for 12 months or usual care	Clinically meaningful benefits in VO2 peak and cardiac reserve after 12 months of exercise training but it did not attenuate functional disability
ONCORE study ClinicalTrials.gov Identifier: NCT03964142 (139)	Ongoing BC patients	CORE program, with measurements of echo parameters, biomarkers and VO2 peak	

This is not an exhaustive list.

BC, breast cancer; CV, cardiovascular; CVD, cardiovascular disease; CORE, cardio-oncology rehabilitation; CRF, cardiorespiratory fitness; VO2, maximal oxygen uptake.

## 9. Socio-economic impact of survivorship care

Lifelong monitoring of all survivors is not feasible due to its unaffordable costs (140, 141). However, even in countries with a National Health System providing free medical care to all citizens, patients of low socio-economic status may not receive optimal care (142). Inexpensive tests, such as an ECG, can be provided as basic screening in survivors of childhood cancers, but more expensive and time-consuming tests should be limited to selected high-risk patients (143). Cancer survivorship also has a socio-economic impact on the patients themselves, as adult CSs may find it difficult to return to work and childhood, adolescent and young adult CSs may have a limited ability to work. The “quality of life” of CSs must be addressed as well. Indeed, it is among the areas of action targeted by the recent European Union in its EU4Health Program, which includes a study of the challenges that prevent CSs from returning to work. The Dutch Childhood Oncology Group's guidelines for long-term follow-up recommend that CSs and those whose malignant disease is in long-term remission attend a survivorship clinic 5 years after diagnosis (144). The needs of CSs can best be addressed in high-quality survivorship care plans, which should consider the stakeholders, their mode of operation and interaction within the care system, and the expectations of CSs. However, such programs are the exception, as, unfortunately, only a minority of CSs are included in a survivorship care plans, due either to the fragmentation of healthcare systems or to a lack of compliance by CSs (145–147). Technology platforms may offer solutions to these and other obstacles that hinder the wider implementation of survivorship care plans (148, 149).

## 10. Conclusions

CSs are an increasing and heterogeneous population with a high burden of CVD. Ever since 2006 when the Institute of Medicine's report “From cancer patient to cancer survivor: Lost in Transition” highlighted the many shortcomings in the CSs' care (150), some progresses have been made, but CSs' care in the post-treatment phase is still fragmented. This is the main reason of the joint effort made by ANMCO (Associazione Nazionale Medici Cardiologi Ospedalieri) and AIOM (Associazione Italiana di Oncologia Medica) in writing a paper that could provide guidance in the management of CSs. An emerging issue on cancer survivorship, the “silver tsunami”, is also analyzed (5).

Given the huge and increasing number of CS, planning a *sustainable* lifelong follow-up is mandatory. A roadmap is suggested whose pillars are a patient-centered approach, a prevention-oriented policy and a multidisciplinary-guided survivorship care plan in a truly *cancer care continuum* perspective. In order to achieve a personalized care of CSs, the multidisciplinary team has to be engaged with different tasks in the pre-treatment, treatment and post-treatment phases. Cardio-Oncologists and Onco-Hematologists lead the pre-treatment phase while Primary Care Provider (PCP), Primary Care Giver (PCG) and the support team (endocrinologists, geriatricians, palliative care providers, psychologists, nephrologists, nutritionists, nurses, social workers) have a complementary helping role. Treatment is the most challenging phase, therefore Cardio- Oncologists, Onco-Hematologists, PCPs, PCGs, and support team all together lead the way in a close collaboration. PCP, PCG and geriatricians are the leaders in charge in the long post-treatment phase getting advices from Cardio-Oncologists and Onco-Hematologists. Throughout the journey there is a compelling need for rehabilitation, that is pre-habilitation, habilitation and post-habilitation in the three different phases. Patients are the core of the survivorship plan and their empowerment make them able to cope with the medical and psychological consequences of cancer and of the oncologic treatments, improving their quality of life. As a matter of facts Quality of life of CSs is an area of action of the European Commission Implementing Decision on the financing of the Programme for the Union's action in the field of health (“EU4Health Programme”). Moreover high-quality survivorship care is urgently needed to ban inequalities. Another important tool to end disparities is digital medicine that has to gain its important role in this process (151, 152).
