# Corrigendum: Cancer survivorship at heart: a multidisciplinary cardio-oncology roadmap for healthcare professionals

**DOI:** 10.3389/fcvm.2023.1309921

**Published:** 2023-10-25

**Authors:** Irma Bisceglia, Maria Laura Canale, Nicola Silvestris, Giuseppina Gallucci, Andrea Camerini, Alessandro Inno, Massimiliano Camilli, Fabio Maria Turazza, Giulia Russo, Andrea Paccone, Raffaella Mistrulli, Leonardo De Luca, Stefania Angela Di Fusco, Luigi Tarantini, Fabiana Lucà, Stefano Oliva, Antonella Moreo, Nicola Maurea, Vincenzo Quagliariello, Giuseppina Rosaria Ricciardi, Chiara Lestuzzi, Damiana Fiscella, Iris Parrini, Vito Racanelli, Antonio Russo, Lorena Incorvaia, Fabio Calabrò, Giuseppe Curigliano, Saverio Cinieri, Michele Massimo Gulizia, Domenico Gabrielli, Fabrizio Oliva, Furio Colivicchi

**Affiliations:** ^1^ Integrated Cardiology Services, Cardio-Thoracic-Vascular Department, Azienda Ospedaliera San Camillo-Forlanini, Rome, Italy; ^2^ Division of Cardiology, Ospedale Versilia, Azienda Usl Toscana Nord Ovest, Lido di Camaiore, Italy; ^3^ Unit of Medical Oncology, Department of Human Pathology in Adulthood and Childhood Gaetano Barresi, University of Messina, Messina, Italy; ^4^ Cardio-oncology Unit, Department of OncoHaematology, IRCCS Referral Cancer Center of Basilicata, Rionero in Vulture (PZ), Italy; ^5^ Department of Medical Oncology, Ospedale Versilia Azienda Usl Toscana Nord Ovest, Lido di Camaiore, Italy; ^6^ Department of Oncology, Sacro Cuore Don Calabria Hospital (IRCCS), Negrar, Italy; ^7^ Department of Cardiovascular and Pulmonary Sciences, Catholic University of the Sacred Heart and Department of Cardiovascular Medicine, Fondazione Policlinico Universitario A. Gemelli IRCCS, Rome, Italy; ^8^ Cardiology Department, National Cancer Institute Foundation (IRCCS), Milan, Italy; ^9^ SC Patologie Cardiovascolari, Azienda Sanitaria Universitaria Giuliano Isontina (ASUGI), Trieste, Italy; ^10^ Department of Cardiology, G. Pascale National Cancer Institute Foundation (IRCCS), Naples, Italy; ^11^ Cardiology Unit, Department of Clinical and Molecular Medicine, Faculty of Medicine and Psychology, Sapienza University of Rome, Rome, Italy; ^12^ Division of Cardiology, San Camillo-Forlanini Hospital, Rome, Italy; ^13^ Clinical and Rehabilitation Cardiology Unit, San Filippo Neri Hospital, ASL Roma 1, Rome, Italy; ^14^ Divisione di Cardiologia, Arcispedale S. Maria Nuova, Azienda Unità Sanitaria Locale- IRCCS di Reggio-Emilia, Reggio Emilia, Italy; ^15^ Cardiologia Interventistica, Utic, Grande Ospedale Metropolitano, Azienda Ospedaliera Bianchi Melacrino Morelli, Reggio Calabria, Italy; ^16^ UOSD Cardiologia di Interesse Oncologico IRCCS Istituto Tumori “Giovanni Paolo II”, Bari, Italy; ^17^ Cardio Center De Gasperis, Azienda Socio Sanitaria Territoriale Grande Ospedale Metropolitano Niguarda, Milan, Italy; ^18^ Unit of Medical Oncology, Papardo Hospital, Messina, Italy; ^19^ Centro Medico Esperia, Porcia (PN), Italy; ^20^ U.O.C. Cardiologia, Ospedale Garibaldi-Nesima, Azienda di Rilievo Nazionale e Alta Specializzazione “Garibaldi”, Catania, Italy; ^21^ Department of Cardiology, Hospital Mauritian Turin, Turin, Italy; ^22^ Department of Interdisciplinary Medicine, School of Medicine, University of Bari Aldo Moro, Bari, Italy; ^23^ Department of Surgical, Oncological and Oral Sciences, Section of Medical Oncology, Palermo University Hospital, Palermo, Italy; ^24^ Department of Oncology and Specialized Medicine, San Camillo-Forlanini Hospital, Rome, Italy; ^25^ Department of Oncology and Hemato-Oncology, University of Milan; Division of Early Drug Development, Istituto Europeo di Oncologia, IRCCS, Milan, Italy; ^26^ Medical Oncology Division and Breast Unit, Senatore Antonio Perrino Hospital, ASL Brindisi, Brindisi, Italy; ^27^ Fondazione per il Tuo cuore- Heart Care Foundation, Firenze, Italy; ^28^ Cardiologia 1- Emodinamica, Dipartimento Cardiotoracovascolare “A. De Gasperis”, Azienda Socio Sanitaria Territoriale Grande Ospedale Metropolitano Niguarda, Milan, Italy

**Keywords:** cancer survivors (CSs), cardiovascular disease (CVD), cancer therapy-related cardiovascular toxicities (CTR-CVT), cardiovascular risk factors (CVRF), reverse cardio-oncology, survivorship care

A Corrigendum on Cancer survivorship at heart: a multidisciplinary cardio-oncology roadmap for healthcare professionals By Bisceglia I, Canale ML, Silvestris N, Gallucci G, Camerini A, Inno A, Camilli M, Turazza FM, Russo G, Paccone A, Mistrulli R, De Luca L, Di Fusco SA, Tarantini L, Lucà F, Oliva S, Moreo A, Maurea N, Quagliariello V, Ricciardi GR, Lestuzzi C, Fiscella D, Parrini I, Racanelli V, Russo A, Incorvaia L, Calabrò F, Curigliano G, Cinieri S, Gulizia MM, Gabrielli D, Oliva F, Colivicchi F. (2023) Front. Cardiovasc. Med. 10:1223660. doi:10.3389/fcvm.2023.1223660

**Incorrect Affiliation**

In the published article, there was an error in affiliation [27]. Instead of “[Division of Cardiology, San Camillo-Forlanini Hospital, Rome, Italy]”, it should be “[Fondazione per il Tuo cuore—Heart Care Foundation, Firenze, Italy]”.

The authors apologize for this error and state that this does not change the scientific conclusions of the article in any way. The original article has been updated.

